# Machine-learning prediction model for acute skin toxicity after breast radiation therapy using spectrophotometry

**DOI:** 10.3389/fonc.2022.1044358

**Published:** 2023-01-06

**Authors:** Savino Cilla, Carmela Romano, Gabriella Macchia, Mariangela Boccardi, Donato Pezzulla, Milly Buwenge, Augusto Di Castelnuovo, Francesca Bracone, Amalia De Curtis, Chiara Cerletti, Licia Iacoviello, Maria Benedetta Donati, Francesco Deodato, Alessio Giuseppe Morganti

**Affiliations:** ^1^ Medical Physics Unit, Gemelli Molise Hospital, Campobasso, Italy; ^2^ Radiation Oncology Unit, Gemelli Molise Hospital, Campobasso, Italy; ^3^ Radiation Oncology, Istituti di Ricovero e Cura a Carattere Scientifico (IRCCS) Azienda Ospedaliero-Universitaria di Bologna, Bologna, Italy; ^4^ Mediterranea Cardiocentro, Napoli, Italy; ^5^ Department of Epidemiology and Prevention, IRCCS Neuromed, Pozzilli, Italy; ^6^ Department of Medicine and Surgery, Research Center in Epidemiology and Preventive Medicine (EPIMED), University of Insubria, Varese, Italy; ^7^ Istituto di Radiologia, Universitá Cattolica del Sacro Cuore, Rome, Italy; ^8^ Department of Experimental, Diagnostic, and Specialty Medicine - DIMES, Alma Mater Studiorum Bologna University, Bologna, Italy

**Keywords:** machine learning, radiation oncology, breast, toxicity, spectrophotometry

## Abstract

**Purpose:**

Radiation-induced skin toxicity is a common and distressing side effect of breast radiation therapy (RT). We investigated the use of quantitative spectrophotometric markers as input parameters in supervised machine learning models to develop a predictive model for acute radiation toxicity.

**Methods and materials:**

One hundred twenty-nine patients treated for adjuvant whole-breast radiotherapy were evaluated. Two spectrophotometer variables, i.e. the melanin (I_M_) and erythema (I_E_) indices, were used to quantitatively assess the skin physical changes. Measurements were performed at 4-time intervals: before RT, at the end of RT and 1 and 6 months after the end of RT. Together with clinical covariates, melanin and erythema indices were correlated with skin toxicity, evaluated using the Radiation Therapy Oncology Group (RTOG) guidelines. Binary group classes were labeled according to a RTOG cut-off score of ≥ 2. The patient’s dataset was randomly split into a training and testing set used for model development/validation and testing (75%/25% split). A 5-times repeated holdout cross-validation was performed. Three supervised machine learning models, including support vector machine (SVM), classification and regression tree analysis (CART) and logistic regression (LR), were employed for modeling and skin toxicity prediction purposes.

**Results:**

Thirty-four (26.4%) patients presented with adverse skin effects (RTOG ≥2) at the end of treatment. The two spectrophotometric variables at the beginning of RT (I_M,T0_ and I_E,T0_), together with the volumes of breast (PTV2) and boost surgical cavity (PTV1), the body mass index (BMI) and the dose fractionation scheme (FRAC) were found significantly associated with the RTOG score groups (p<0.05) in univariate analysis. The diagnostic performances measured by the area-under-curve (AUC) were 0.816, 0.734, 0.714, 0.691 and 0.664 for IM, IE, PTV2, PTV1 and BMI, respectively. Classification performances reported precision, recall and F1-values greater than 0.8 for all models. The SVM classifier using the RBF kernel had the best performance, with accuracy, precision, recall and F-score equal to 89.8%, 88.7%, 98.6% and 93.3%, respectively. CART analysis classified patients with I_M,T0_ ≥ 99 to be associated with RTOG ≥ 2 toxicity; subsequently, PTV1 and PTV2 played a significant role in increasing the classification rate. The CART model provided a very high diagnostic performance of AUC=0.959.

**Conclusions:**

Spectrophotometry is an objective and reliable tool able to assess radiation induced skin tissue injury. Using a machine learning approach, we were able to predict grade RTOG ≥2 skin toxicity in patients undergoing breast RT. This approach may prove useful for treatment management aiming to improve patient quality of life.

## Introduction

The role of post-operative radiotherapy is today considered crucial in breast cancer (1). Over 70% of breast cancer patients receive radiation therapy, which decreases local recurrence rates and improves long-term survival (2). Because survival from breast cancer continues to improve in the last years (3), the assessment of quality of life and adverse effects caused by irradiation has become more and more relevant (4). In particular, acute toxicity, including breast erythema and desquamation (skin loss), has a major role in side effects. For some patients, desquamation can cause significant patient morbidity, worsening of the cosmetic outcome following surgery and also the interruption of the treatment course thereby decreasing the dose and potentially increasing the risk of local recurrence (5).

The predictive role of clinical and treatment risk factors for acute breast radiation toxicity has been explored by several studies (6–10). However, prediction models able to score individual patient toxicity risk have shown limited success. Mbah et al. (11) aimed to identify the main causes underlying the failure of prediction models for radiation therapy toxicity to replicate in breast cancer patients. The authors reported that the overfitting and the cohort heterogeneity must be considered the two main causes of replication failure of prediction models across cohorts. If cross-validation and bootstrapping may cope with overfitting, it was highlighted that any reliable predictive model for radiation therapy toxicity requires robust strategies to deal with cohort heterogeneity.

Recent studies have demonstrated the capability of machine learning (ML) to develop predictive models for radiation toxicities in different cancers (12). Nowadays, at the best of our knowledge only two studies employed In particular, a few studies tried to implement ML-based models to predict breast skin toxicity after breast radiotherapy. Saednia et al. (13) proposed a novel method for detecting the increase in body surface temperature caused by radiation dermatitis. Thermal images of the irradiated breast of ninety patients taken during the treatment course were used to build a ML model. The thermal markers at the fifth treatment fraction were found predictive of acute skin toxicity with a prediction accuracy of 0.87. Recently, Aldraimli et al. (14) developed several clinical prediction models for acute breast desquamation after whole breast external beam radiation therapy in the prospective multicenter REQUITE cohort study. After optimization, the random forest algorithm was found the best model, able to classify patients with acceptable performance in the validation cohort (AUC = 0.77). Feng et al. (15) developed a novel quantitative ML tool for prediction of grade ≥ 2 dermatitis before radiotherapy by using data encapsulation screening and multi-region dose-gradient-based radiomics techniques, in addition to clinical and dosimetric parameters. Using data of 214 patients, a combination of 20 radiomics features and 8 clinical and dosimetric variable achieve an AUC of 0.911 in the validation dataset. Lastly, Li et al. (16) were able to develop a ML framework by integrating multi-region dose gradient correlated-radiomics features from planning CT and clinical and dosimetric factors. A random forest model trained with 10 radiomics features, 3 dosimetric and 6 clinical variables achieved a performance of AUC equal to 0.946 in predicting severe radiation dermatitis after radiotherapy.

Spectrophotometry is an alternative quantitative approach to directly and objectively measure the changes of skin surface characteristics that may be associated with radiation-induced skin toxicity. Yoshida et al. (17) investigated the use of spectrophotometry to study skin discoloration as a marker for subcutaneous-tissue fibrosis during breast-cancer treatments. The authors reported significant differences between the treated and contralateral breasts among all patients, with a 27% and 23% mean increase in melanin and erythema (p < 0.001), respectively, with all parameters correlated with toxicity scores. Schmeel et al. (18) performed photospectrometric skin readings in 140 patients aiming to objectively determine frequency and severity of acute radiation-induced skin reactions during whole breast irradiation. The photospectrometric measurements reported both decreased erythema severity (p = 0.008) and hyperpigmentation (p = 0.002) in the hypofractionation arm, according to physician-rated observations.

All these quantitative features may then be used as imaging biomarkers in a ML framework to develop predictive tools for radiation-induced skin toxicity. Although a few previous studies have proposed the use of spectrophotometry to monitor skin changes during radiation therapy, the potential for employing spectrophotometric features as imaging biomarkers for prediction of radiation-induced dermatitis using ML strategies is still unknown and requires additional research.

Based on the aforementioned arguments, the goal of this paper was to develop a predictive model for acute breast radiation toxicity using different ML classification models based on clinical and spectrophotometric variables. We aimed to measure photo-spectrometric characteristics of the irradiated skin in patients with breast cancer and we hypothesized that radiation-induced skin toxicity is associated with a variation of skin characteristics as melanin and erythema.

## Material and methods

### Study design

The present study is a sub-analysis of a double-blind randomized placebo-controlled trial (ATHENA project) that aimed to investigated the potential beneficial effect of anthocyanins supplementation on skin toxicity after breast radiotherapy (19). This study was registered in clinicaltrial.gov with identifier NCT02195960 and approved by the institutional research ethics board. An informed consent form was signed by participants before enrollment.

### Patient selection

A total of 129 patients with breast cancer undergoing radiation therapy after surgery were enrolled. Inclusion criteria were the type of tumor (invasive carcinoma of the breast), surgical treatment (lumpectomy or quadrantectomy) and axillary staging. Exclusion criteria were: women with suspected or confirmed residual disease after surgery, non-epithelial breast malignancies, proven multicentric carcinoma (invasive or ductal carcinoma in situ) in more than one quadrant or separated by four or more centimeters, Paget’s disease of the nipple, synchronous bilateral invasive or non-invasive breast cancer, breast implants, prior breast or thoracic RT for any condition, collagen vascular disease, specifically dermatomyositis with a creatine phosphokinase level above normal or with an active skin rash, systemic lupus erythematosus, or scleroderma, pregnancy or lactation at the time of proposed randomization, psychiatric or addictive disorders or other conditions that, in the opinion of the investigator, would preclude the patient from meeting the study requirements.

Demographic and clinical characteristics are presented in **Table 1**.

**Table 1:** Patient characteristics.

Categorical variable	Patients (%)
Number of patients	29 (100)
Acute Skin Toxicity	
No toxicity	49 (38.0)
Grade 1	46 (35.7)
Grade 2	32 (24.8)
Grade 3	2 (1.5)
Fractionation regimen	
50-60 Gy/25 fx	80 (62.0)
40-44 Gy/16 fx	49 (38.0)
Chemotherapy	
No	69 (53.5)
Yes	60 (46.5)
Hormone therapy	
No	118 (91.5)
Yes	11 (8.5)
Laterality	
Left	60 (46.5)
Right	69 (53.5)
Quadrant	
Upper, outer	57 (44.2)
Upper, inner	23 (17.8)
Lower, outer	12 (9.3)
Lower, inner	23 (17.8)
Central	14 (10.9)
Continuous variable	Median (range)
Age (years)	62 (45-89)
BMI	26.4 (19.0-47.1)
PTV2 (cm^3^)	689 (118-2833)
PTV1 (cm^3^)	95 (4-499)

### Treatment

All patients were simulated in the supine position with the C-Qual TM Breastboard system (Civco Medical Solutions, Kalona, IA, USA), with the ipsilateral arm placed above their heads. Computed tomography imaging was performed with slice thickness acquisition of 3mm extending from the larynx to the upper abdomen. Two clinical target volumes (CTVs) were defined, including the breast (CTV2) and the tumor bed (CTV1), the latter delineated on the basis of preoperative and operative reports and including the surgical clips and/or any surgery induced changes considered to be a part of the lumpectomy cavity (hematoma or seroma). The corresponding planning target volumes (PTV2 and PTV1) were generated by a uniform expansion of the CTVs by 5mm, restricted 3mm from external body.

Planning was performed using a tangential hybrid IMRT technique, consisting of a conventional tangential-field plan plus an inverse-planned IMRT plan (20, 21). The prescription for conventional planning (80% of total dose) was associated with a pair of open medial and lateral beams (with heart and lung MLC blocks). The IMRT prescription (20% of total dose) was associated with two step-and-shoot beams, optimized by inverse planning in the Oncentra Masterplan treatment planning system. A maximum of 5 segments per beam was allowed to reduce treatment time.

Patients were treated using two different treatment schedules according to low (group 1) or moderate-high (group 2) risk of recurrences. Patients in group 1 were treated using an hypofractionated daily dose of 2.50 Gy and 2.75 Gy to a total of 40 Gy and 44 Gy to PTV2 and PTV1 in 16 fractions, respectively. Patients in group 2 were treated using a conventional daily dose of 2.0 Gy and 2.4 Gy to a total of 50 Gy and 60 Gy to PTV2 and PTV1 in 25 fractions, respectively (22). The two groups were not equal, with 49 patients receiving hypofractionation and 80 receiving conventional fractionation.

The planning objectives for both PTVs were as follow: 98% of the PTVs should receive more than 95% of each prescription dose and no more than 2% of the PTVs should exceed 107% of prescription doses. The dose limits for the OAR mean doses were as follows: ipsilateral lung,<8 Gy; contralateral lung,<2 Gy; heart,<4 Gy; and contralateral breast,<2 Gy. During plans optimization we also followed the specific QUANTEC (Quantitative Analyses of Normal Tissue Effects in the Clinic) suggestions for the heart and lung: the lung volume receiving more than 20 Gy should not exceed 25% (V20Gy<25%) (23) and the heart volume receiving more than 25 Gy should not exceed 10% (V25 Gy< 10%) (24).

### Spectrophotometry: Melanin and erythema indexes

Skin injury was quantified by narrow band spectrophotometer measurements of melanin and erythema indices. The used spectrophotometer was the Mexameter MX (Courage+Khalaza Electronic GmbH, Germany). The Mexameter probe emits light of three wavelengths: 568 nm (green light), 660 nm (red light), and 880 nm (infrared light). The melanin index (I_M_) is based on the ratio of the intensities of the infrared (Sinfr) and red (Sred) light reflection and it is defined as:


$${\text{I}}_{M}\;=\;\log_{10}\left(\frac{S_{\inf r}}{S_{red}}\right)$$

Similarly, the erythema index (I_E_) is based on the ratio of the intensities of the red (S_red_) and green (S_green_) light reflection and it is defined as:


$${\text{I}}_{E}\;=\;\log_{10}\left(\frac{S_{red}}{S_{green}}\right)$$

These definitions provide a broad-scale values (0-999) for melanin and erythema, in order to detect even smallest changes in color (25).

All patient underwent spectrophotometer measurements in supine position before the beginning of radiation therapy (T0), at the end of therapy (T1), at 1 month (T2) and 6 months (T3) from the end of therapy.

### Candidate covariates

Additional clinical and demographic variables were gathered from the electronic medical records, including: age, cancer diagnosis, clinic-pathologic characteristics of the tumor, surgery details, RT treatment information, administration of adjuvant chemotherapy (y/n), type of adjuvant chemotherapy, local treatment (whole breast only) versus loco-regional irradiation (involving the regional lymph nodes) and menopausal status. The variables used for the algorithm development are reported in **Table 1**.

### Clinical assessment and outcomes

The acute skin toxicity was registered according to the RTOG scoring system (26) in the following groups: Grade 1: Follicular, faint or dull erythema/epilation/dry desquamation/decreased sweating; Grade 2: Tender or bright erythema, patchy moist desquamation/moderate edema; Grade 3: Confluent, moist desquamation other than skin folds, pitting edema; Grade 4: ulceration, hemorrhage, necrosis. The occurrence of acute toxicity in patients was converted into a binary outcome: 0 for patients who experienced grade ≤ 1 (zero or one) and 1 for patients who experienced grade ≥ 2 (two or three) acute toxicity.

### Machine-learning modeling

The patient’s dataset was randomly divided with the Holdout method into training and testing sets used for model development and cross-validation. The training and testing set included 97 and 32 patients, respectively (i.e. 75%/25% split).

Several ML models were developed for binary classification of acute toxicity. ML modeling included two phases. In the first phase, we prevented model overfitting using a multistage feature selection method. First, we assessed the pairwise feature interdependencies using the Spearman rank correlation coefficient, with the goal to identify the functional dependencies between features. The association between each covariate and skin acute toxicity was evaluated with a univariate analysis using the Mann-Whitney U-test, able to evaluate the difference between two independent group populations. A stepwise backward elimination approach was subsequently used for the remaining features, i.e. each variable was considered for subtraction from the set of explanatory variables based on the Akaike information criterion (AIC) (27). AIC is a model selection criterion used to penalize the models for which adding new explanatory variables does not supply sufficient information to the model. The aim is to minimize the AIC, defined as:


AIC=2logL(M)+2K

where logL(M) is the maximized log likelihood for the fitted model, M is the sample size and K is the number of covariates including an intercept.

The surviving variables were finally used to build the models for binary classification of acute toxicity, including logistic regression (LR), support vector machine (SVM), and classification and regression tree analysis (CART).

LR is a classical algorithm that is usually used for binary classification tasks (28). Briefly, this model calculates the class membership probability for one of the two categories in the dataset (0 or 1) using a logistic equation:


$${\text{p}}_{i}\;=\;\frac{e^{\left(\beta_{0}+\beta \cdot x_{i}\right)}}{1+e^{\left(\beta_{0}+\beta \cdot x_{i}\right)}}$$

where x_i_ is the input value and β are the regression coefficients. In order to provide a probability that should vary from 0 to 1, the equation can be linearized by the logit transformation


$${\text{logit(p}}_{\text{i}}\text{) = ln}\left(\frac{{\text{p}}_{i}}{1-{\text{p}}_{i}}\right)\;=\;\beta_{0}+\beta \cdot x_{i}$$

where the logistic unit (logit) is on the left-hand side.

Since the logistic regression predicts probabilities, the likelihood function can be used. Therefore, for each training data point x, the predicted class is y. Probability of y is either p if y=1 or 1-p if y=0. The likelihood can be written as:


$$\text{L(}\beta_{0},\beta \text{) = }\overset{N}{\underset{1}{\Pi }}p{\left(x_{i}\right)}^{y_{i}}(1-p{\left(x_{i}\right)}^{1-y_{i}}$$

that can be transformed as:


$$\text{ln(L) = }\sum_{1}^{\text{N}}\left[{\text{ln(1-p}}_{\text{i}}{\text{)+y}}_{\text{i}}\left(\frac{{\text{p}}_{i}}{1-{\text{p}}_{i}}\right)\right]$$

or, rewritten in term of the “logistic loss” function L_log_:


$${\text{L}}_{\text{log}}\;=\;\ln(L)\;=-N\sum_{1}^{\text{N}}\left[-\ln(1+e^{\left(\beta_{0}+\beta \cdot x_{i}\right)})]+y_{i}(\beta_{0}+\beta \cdot x_{i}\right)$$

A penalty component called the L2 norm was then added to the logistic loss function in order to prevent overfitting. This factor effectively shrinks the estimates of the coefficients toward zero. The new loss function is:


$${\text{L}}_{\text{log}}+\lambda \sum_{1}^{\text{p}}\beta_{\text{j}}^{2}$$

where j is the number of coefficients in the model and Λ is a regularization parameter to be manually tuned. This penalized loss function is also called “Ridge regression”. A 5-fold cross-validation in the training set was performed to determine the optimal value of the λparameter. The goodness of the LR model fit was evaluated by the Hosmer–Lemeshow test, calculating the agreement between the the observed and expected event rates in population subgroups.

SVM-based classification models are one of the most popular supervised classification algorithms. For a given set of data from two groups of patients, the SVM algorithm tries to find the maximum hyperplane between the two classes in order to maximize its distance to the nearest data points on each side (the so-called support vectors. If the two samples are not linearly separable, various kernel functions can be used to transfer them to a higher-dimensional space where they can be separated more easily. In our analysis, we used four different kernel types in this study: linear, power, sigmoid, and radial basis function (RBF) kernels. After a tuning phase to acquire the best classification results, the cost C, an internal training process parameter, was set to 1 to increase the margin distance between the hyperplane and the closest samples in both classes.

Lastly, we built a classification and regression tree analysis (CART) to visually stratify patients into the toxicity risk groups. The CART model is represented as a binary tree, where each root node stands for a single input variable and a split point on that feature. A prediction-making output variable is contained in the tree’s leaf nodes. The Gini impurity (GI) index was used to determine the best splits:


$$GI = 1-\sum_{i=1}^{n}{\text{p}}_{i}^{2}$$

where p_i_ is the fraction of items in the class i.

The models were cross-validated using 5-fold cross-validation five repeated holdout evaluations, each time with a different random partition of the data, with the goal to reduce both the variance of the cross-validation results and the unlikely possibility of getting too optimistic results in only one run. This resampling technique maintains a balanced distribution of both classes in each fold by randomly dividing each feature dataset into five subsets of samples of equal size. Then, five models were trained and tested; each of the five folds was tested once, and the model was trained using the other four folds. The procedure was performed five times with the goal to reduce both the variance of the cross-validation results and the unlikely possibility of getting too optimistic results in only one run.

The performance of the models was assessed using class-specific accuracy, precision, recall and F-measure evaluation metrics. The accuracy is defined as the proportion of correct predictions (both true positives and true negatives) among the total number of cases examined. The precision is defined as the number of true positive results divided by the number of all positive results, including those not identified correctly (i.e. it is the positive predictive value). A low precision indicates a large number of false positives. The recall is defined as the number of true positive results divided by the number of all samples that should have been identified as positive (i.e. it is the sensitivity in binary classification or true positive rate). A low recall indicates many False Negatives. The F-score is the harmonic mean of the precision and recall. These indexes range from 0 to 1, with higher values indicating better classification performance.

**Figure 1:**
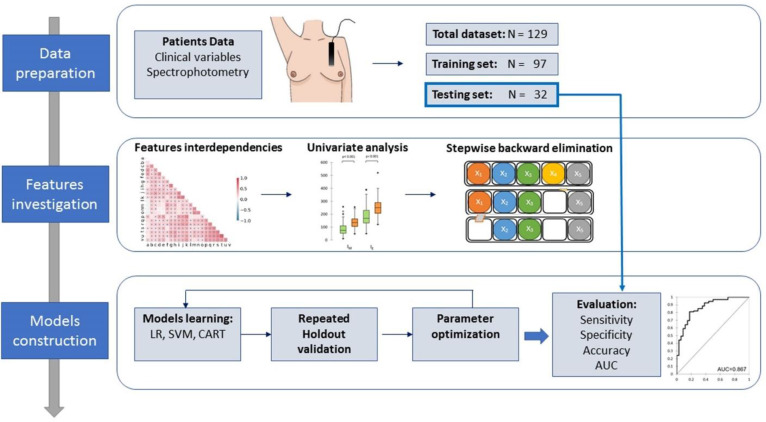
Schematic workflow and methodology of the study.

**Figure 1** shows the flow diagram for the model development.

### Statistical analysis

All statistical analyses were performed using the XLSTAT statistical packages (Addinsoft, New York, USA).

## Results

### Patient characteristics

Of the 129 patients enrolled in this study (median age 62, range 45-89), 34 (26.4%) presented RTOG ≥ 2 acute toxicity at the end of their treatment.

### Spectrophotometry measurements

**Figure 2:**
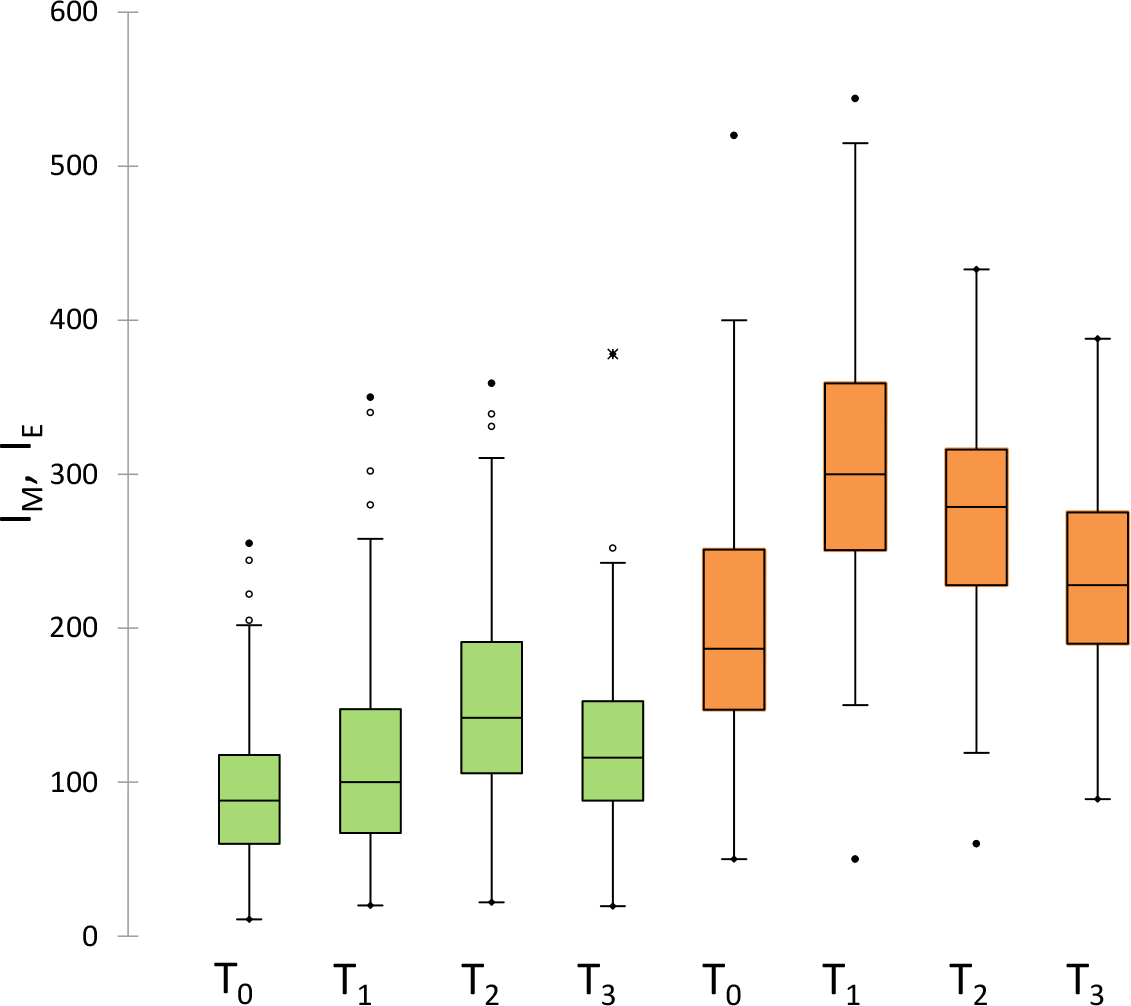
Measured values of melanin (green) and erythema (orange) indexes before (T0) and after different times (T1, T2 and T3) after radiotherapy. Data are reported as box-and-whisker plots.

**Figure 2** shows the values of melanin (green) and erythema (orange) indexes before (T_0_) and after different times (T_1_, T_2_ and T_3_) after radiotherapy. Data are reported as box-and-whisker plots. Melanin significantly increased from T_0_ to T_1_ and to T_2_ by about 14% and 60%, respectively, and decreased in the following observation times towards values similar to T_0_ at one year (T_3_). Similarly, the erythema index significantly increased from T_0_ to T_1_ by about 60%, and decreased in the following observation times.

### Variables selection

In univariate analysis (Mann-Whitney U-test), the two spectrophotometric variables at time T_0_ (I_M,T0_ and I_E,T0_), together with the BMI, PTV1, PTV2 and the dose fractionation scheme (FRAC) were found to be significantly associated with the two RTOG groups (p<0.05).

Detailed diagnostic accuracy statistics for the five quantitative covariates significantly associated with the toxicity score is reported in **Table 2**.

**Table 2:** Detailed diagnostic accuracy statistics for the five quantitative covariates significantly associated with the toxicity score.

	Area Under Curve (AUC) (CI95%)	Sensitivity	Specificity	Positive predictive value (PPV)	Negative predictive value (NPV)	Accuracy
Spectrophotometry						
I_M_	0.816 (0.738-0.891)	0.882	0.684	0.500	0.942	0.736
I_E_	0.734 (0.637-0.831)	0.765	0.705	0.481	0.893	0.721
Clinical						
PTV1	0.691 (0.583-0.799)	0.676	0.684	0.434	0.855	0.682
PTV2	0.714 (0.611-0.817)	0.676	0.726	0.469	0.863	0.713
BMI	0.664 (0.560-0.767)	0.853	0.467	0.377	0.894	0.573

**Figure 3:**
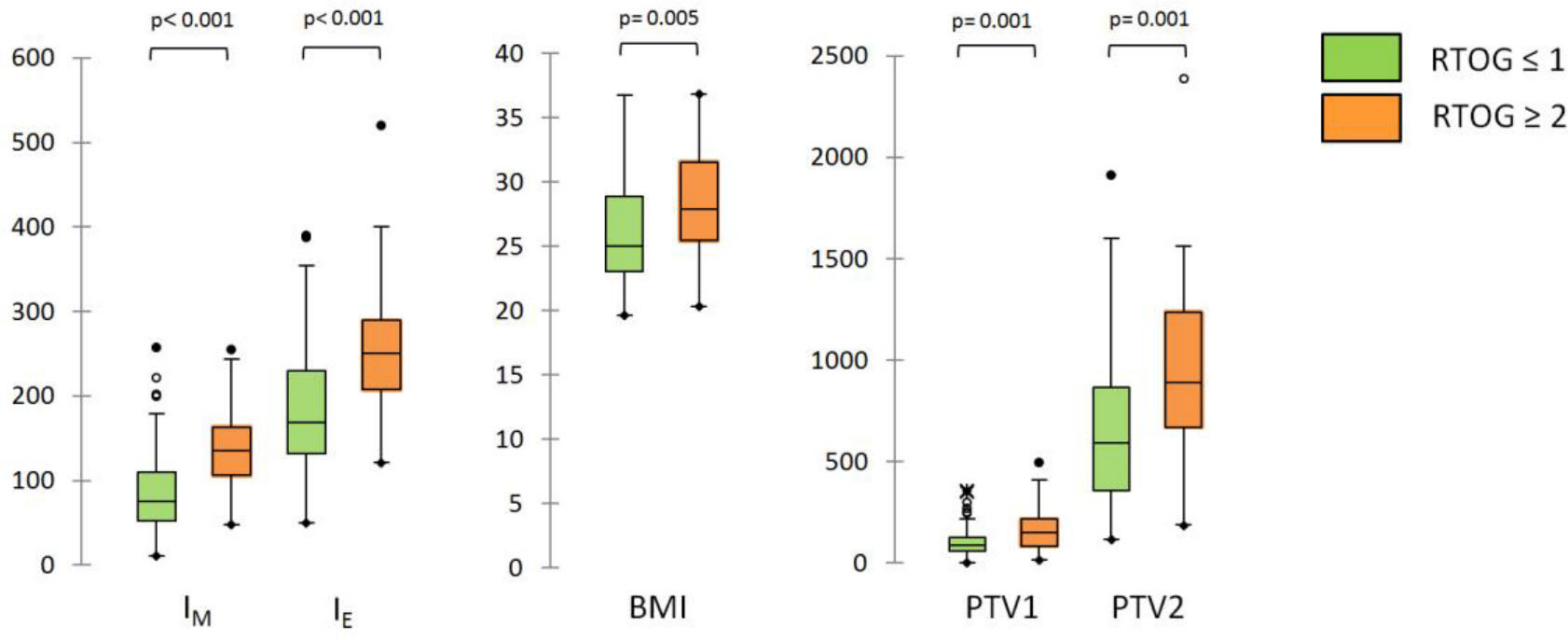
Box-and-whisker plots for I_M,T0_, I_E,T0_ and the three continuous variables (BMI, PTV1 and PTV2) associated with RTOG toxicity classification at univariate analysis.

The box-and-whisker plots for I_M,T0_, I_E,T0_ and the three continuous variables associated with RTOG toxicity classification are shown in **Figure 3**; the diagnostic performance measured by AUCs were 0.816, 0.734, 0.714, 0.691 and 0.664 for I_M_, I_E_, PTV2, PTV1 and BMI, respectively.

### Machine learning modeling and performance

The classification performances of LR, SVM and CART models are reported in **Table 3**.

**Table 3:** Performances of the LR, SVM and CART classifier for the training and validation datasets.

	Machine learning models					
	LR	SVM				CART
		*Linear*	*Sigmoid*	*Power*	*RBF*	
Training set						
Accuracy	0.831 (0.807-0.855)	0.845 (0.835-0.855)	0.742 (0.732-0.752)	0.874 (0.865-0.883)	0.898 (0.889-0.906)	0.866 (0.851-0.881)
Precision	0.845 (0.824-0.865)	0.856 (0.844-0.868)	0.776 (0.764-0.788)	0.860 (0.850-0.870)	0.886 (0.877-0.895)	0.869 (0.849-0.889)
Recall	0.937 (0.926-0.948)	0.937 (0.929-0.945)	0.801 (0.793-0.809)	0.968 (0.961-0.975)	0.986 (0.980-0.992)	0.962 (0.943-0.981)
F1 score	0.889 (0.873-0.904)	0.895 (0.888-0.902)	0.788 (0.781-0.795)	0.911 (0.905-0.917)	0.933 (0.928-0.939)	0.913 (0.902-0.923)
Testing set						
Accuracy	0.801 (0.752-0.851)	0.805 (0.761-0.849)	0.722 (0.678-0.766)	0.825 (0.788-0.862)	0.843 (0.808-0.878)	0.829 (0.761-0.897)
Precision	0.867 (0.838-0.895)	0.841 (0.798-0.884)	0.732 (0.689-0.775)	0.822 (0.785-0.859)	0.841 (0.807-0.876)	0.855 (0.793-0.917)
Recall	0.875 (0.829-0.921)	0.882 (0.863-0.901)	0.791 (0.772-0.810)	0.961 (0.945-0.977)	0.981 (0.966-0.996)	0.931 (0.855-1.000)
F1 score	0.871 (0.838-0.901)	0.861 (0.835-0.887)	0.760 (0.734-0.786)	0.886 (0.864-0.908)	0.906 (0.885-0.925)	0.891 (0.836-0.946)

Classification performances reported precision, recall and F1-values greater than 80% for all models. The SVM classifier using the RBF kernel had the best performance, with accuracy, precision, recall and F-score equal to 89.8%, 88.7%, 98.6% and 93.3%, respectively.

**Figure 4:**
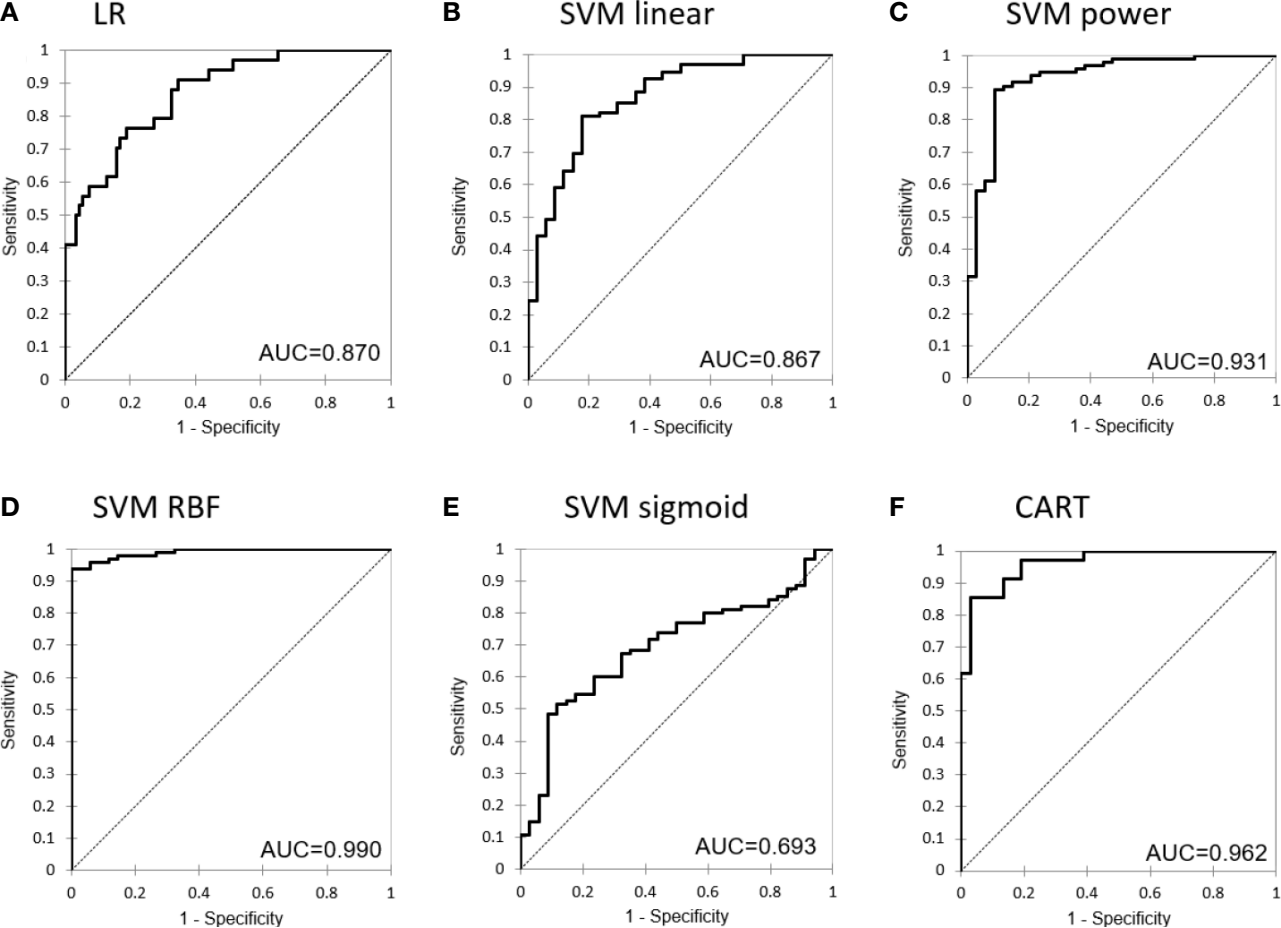
Receiver operating characteristic (ROC) curve for the different machine learning models: **(A)** Logistic Regression, **(B)** Support Vector Machine with linear kernel, **(C)** Support Vector Machine with power kernel, **(D)** Support Vector Machine with RBF kernel, **(E)** Support Vector Machine with sigmoid kernel and **(F)** Classification and Regression Tree.

**Figure 4** shows the receiver operating characteristics (ROC) curves of the different models for the testing set.

**Figure 5:**
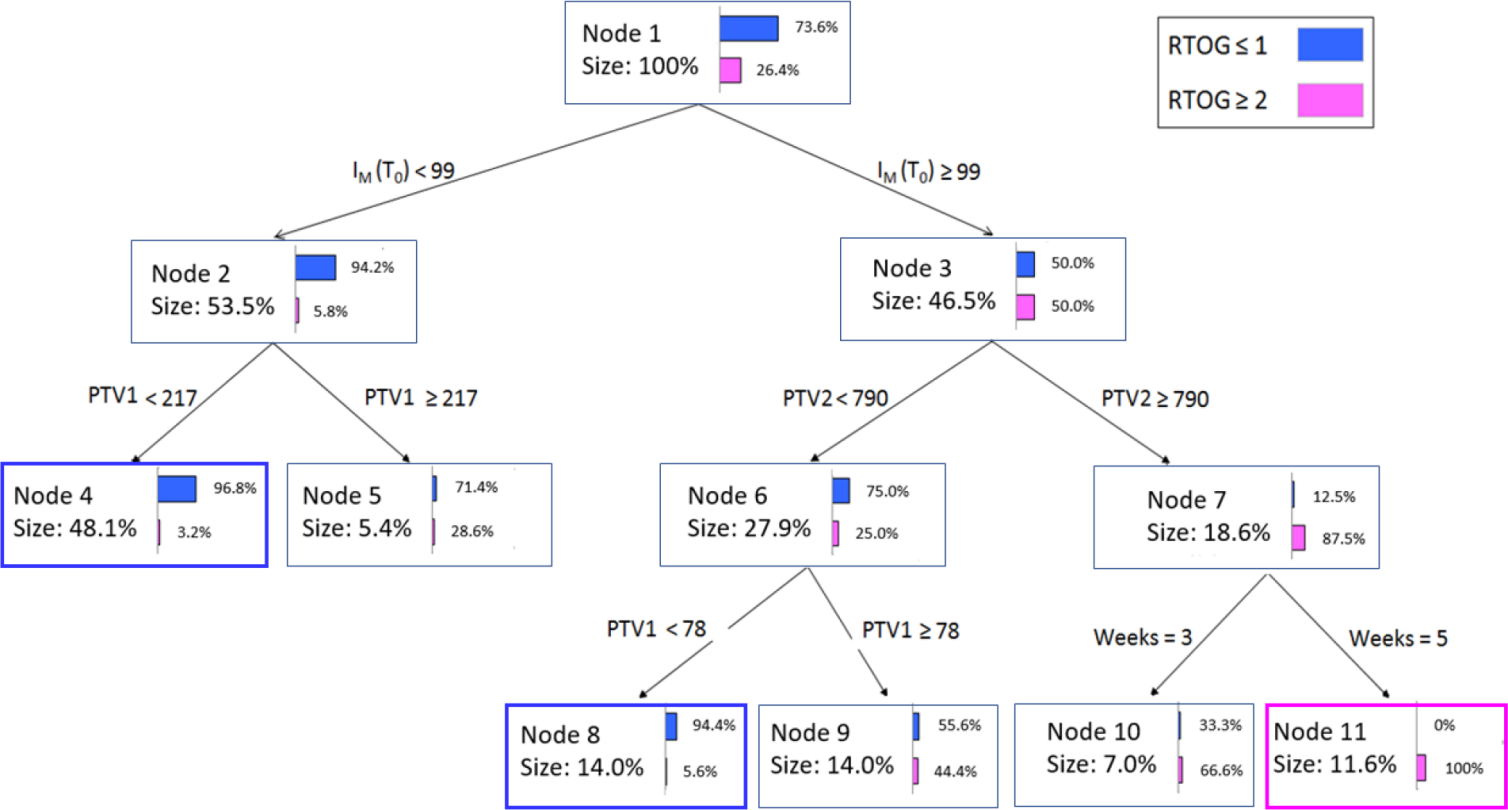
Classification and Regression tree analysis (CART) for the most significant variables.

The CART classification tree for the most informative variables is displayed in **Figure 5**. Each node reports the following information: (a) the total number of objects, (b) the corresponding percentage, (c) the improvement corresponding to the number of observations in the node times, (d) the purity, which shows what percentage of objects at this node fall into the category that dominates the dependent variable, (e) the split variable, and (f) the value or intervals of the latter.

Node 1 illustrates the initial patient distribution prior to the application of any criteria. Nodes 2 and 3 show the results of adding the I_M,T0_ variable as the initial criterion for decisions. The I_M,T0_< 99 cutoff value relocates most of the patients to node 2 with a purity index of 94.2%, e.g., almost all patients do not present RTOG ≥ 2 grade acute toxicity. The remaining patients (having I_M,T0_ ≥ 99 value) are equally associated with or without RTOG ≥ 2 grade toxicity in node 3, and are additionally separated based on a second criterion., e.g., the PTV2 volume. At this second decision level, PTV2 values greater than 790 cm^3^ relocate patients to the RTOG ≥ 2 grade with 87.5% purity, and so on. **Figure 5** illustrates how the rules obtained by the algorithm can be expressed in natural language.: “At the beginning of treatment, if the melanin index I_M,T0_< 99 then patients will not experience RTOG ≥ 2 grade acute toxicity in 94.2% of cases. Furthermore, for these patients, if PTV1<217 cm^3^ then the probability increases to 96.8%”. Similarly, at node 3 and 7, “if I_M,T0_ ≥ 99 and PTV2 > 790 cm^3^ then patients will belong to the RTOG ≥ 2 grade group in 87.5% of cases”.

## Discussion

The knowledge of patient and treatment factors contributing to acute toxicities after whole-breast radiotherapy is a crucial point to guide treatment decisions, target supportive care and inform patients. If patients who are inherently most sensitive to radiation could be identified prior to treatment, early-intervention personalized therapeutics pathway becomes possible.

A recent systematic review and meta-analysis of 38 studies composed of 15,623 breast cancer patients reported considerable heterogeneity in the evaluation of acute radiation dermatitis and in patient- and treatment related risk factors (29). The proportion of patients with acute radiation dermatitis of grade 2 or higher after radiotherapy ranged from 10% to 76%, with an average of 34.3% and a median of 28.4%. Hypo-fractionated RT was shown to cause the same or less skin toxicity than conventional fractionation and has been adopted by many cancer centers. IMRT also demonstrated consistently better dose homogeneity and decreased skin toxicity relative to wedge-based treatments, and is now standard protocol in centers with the appropriate resources. Four factors were significantly associated with acute radiation dermatitis: body mass index (BMI) ≥25 kg/m^2^, large breast volume, smoking habits and diabetes. Borm et al. (30) evaluated skin toxicity during modern 3D-CRT radiotherapy in a 255-patient cohort. 42.4% of the patients developed grade I, 55.7% grade II and 2% grade III skin toxicities. On univariate analysis breast size correlated significantly with the incidence of skin toxicity. Ciammella et al. (10) evaluated the toxicity and cosmetic outcome in breast cancer patients treated with adjuvant hypo-fractionated radiotherapy in a 212-patient cohort. 16% of patients experienced no acute toxicity, according to the RTOG criteria, while 68% and 15% developed grade 1 and grade 2 acute skin toxicity, respectively. Parekh et al. (31) evaluated the rate of grade ≥2 dermatitis in patients with a high BMI who were treated to the breast or chest wall to understand the role of radiation target, fractionation regimen, and BMI. Grade ≥2 dermatitis was 31.4% among the entire cohort, with significant differences between chest wall and whole breast treatments (48% *vs* 27.8%; p = .007). In the present study, 49 (38.0%) patients experienced no acute skin toxicity. 46 (35.7%), 32 (24.8%) and 2 (1.5%) patients experienced grade 1, grade 2 and grade 3 acute skin toxicities, respectively. These findings are similar to those obtained by Parekh et al. (31).

So far, acute toxicity grade was obtained by qualitative evaluations with visual inspection or palpation. If this approach may be sufficient in clinical routine, the lack of reliable and quantitative assessment was shown to be prone to significant biases due to intra and inter-evaluator differences (32). Consequently, reliable and quantitative measurements are needed, particularly in the context of new therapeutic strategies, in which even minimal differences in dermatitis are of interest (33). A potential technical quantitative approach to assess acute toxicity is spectrophotometry. Back between 1972 and 1985, Turesson et al. (34) already aimed to identify risk factors for RID associated with breast electron irradiation using reflectance spectrophotometry. Spectrophotometry was then applied to objectify skin-color alterations and radiation dermatitis in breast radiotherapy in two recent investigations (17, 18).

Following the aforementioned suggestions, we investigated the potential of spectrophotometry-based ML methods as a reliable tool to quantify skin-color alterations in conventional and hypofractionated breast cancer treatments. To our knowledge, our study is the first to assess breast acute toxicity using spectrophotometric metrics as inputs variables of supervised ML models. A major finding of the present study is the demonstration that the pre-treatment values of melanin and erythema indexes may serve as predictive biomarkers for radiation-induced acute skin toxicity. Both indexes are markers of skin discoloration. Melanin is a pigment produced by membrane-bound melanosomes in the epidermis’ basal layer. Erythema refers to skin redness caused by capillary dilatation and may indicate the onset of telangiectasias. Our results report that patients presenting RTOG ≥ 2 exhibited higher melanin and erythema indexes values before the beginning of treatment compared with those with RTOG ≤ 1 score. This is a quite unexpected finding, based on the wrong assumption that patients with light skin pigmentation will be associated to more severe skin toxicity than patients with darker skin pigmentation. Indeed, a few researchers already highlighted a dependence of radiation-induced skin toxicity severity on skin pigmentation. Wright et al. (35) demonstrated that ethnicity is a risk factor for radiation-induced skin toxicity. In particular, the authors reported that at RT completion, moist desquamation was more common in black patients (73.1% *vs* 47.6%, respectively, p=.023) and on multivariate analysis, the effects of black race (odds ratio [OR] = 7.46, p=.031) remained a significant risk factor for moist desquamation. The reason for these behaviors is still unclear. A few published data (36) reported that genetic mutations associated with the development of acute toxicity are more commonly found in black patients. In particular, the authors reported that the sequence variants located in the ATM gene, having a significantly greater incidence in African women, may predict for adverse radiation responses in breast cancer patients. Another suggestion is based on the action of free radicals generated by radiation on melanin (37). It has been reported that the melanocytes, the cells that produce melanin, are more vulnerable to oxidative stress than skin cells types, such as keratinocyts and fibroblasts (37). The exposure to UV or ionizing radiation can impair the syntheses of melanin and cause skin damage. Although melanin can prevent oxidative DNA damage in melanocytes and keratinocytes, *in vitro* studies demonstrated that reactive oxygen species involved in melanin synthesis can exacerbate DNA damage (38). As a result of the increased rate of melanin, darker skin may be more sensitive to DNA damage from radiation compared to lightly pigmented skin.

Other findings indicated that hypofractionation and the breast and boost cavity volumes were the predictive clinical variables for acute toxicity in our cohort. With respect to hypofractionation, our results are in strong agreement with the recent literature. A recent randomized, non-inferiority, phase III trial reported that hypofractionation (43.5 Gy over 15 fractions) was equally effective in terms of locoregional recurrence, overall survival, and disease-free survival of standard fractionation (50 Gy over 25 fractions) but with a significant reduction of grade 3 acute skin toxicity (3% *vs*. 8%, p< 0.001) (39). Similarly, a large multicenter study on 2309 patients reported significantly higher skin reaction (moist desquamation, 28.5% *vs* 6.6%, p<0.001; grade ≥2 dermatitis, 62.6% *vs* 27.4%, p<0.001) in patients treated with conventional fractionation with respect to those treated with hypofractionation (40). Our results are consistent with these studies, suggesting that an hypofractionation regimen could reduce the risk of radiation dermatitis compared with that in conventional regimen. We also found the breast and boost volumes are positive predictors of more severe toxicity, with a significantly increased risk in larger breasts and boost volumes. Again, this is an expected result because breast volume has been demonstrated to be a predictor of dermatitis in two randomized clinical trials (8, 41). The association between the risk of dermatitis and breast volume is most likely caused by an increase of dose inhomogeneity within the breast, the abrasive effect of friction within skin folds and the bolus effect in the inframammary, skin folds, and axillary regions.

Using these two spectrophotometric and three clinical top ranked features, we trained machine learning models using LR, SVM and CART analysis methods. The best performance was attained by the SVM classifier using the RBF kernel, which was also successful in producing the hyperplane and maximizing the space between the support vectors. SVM reported the higher accuracy and F-score values, able to correctly classify 70 out 71 grade 0-1 patients and 21 out 26 grade 2-3 patients in the training dataset. In addition, given of its well-known interpretability in terms of variable thresholds, we developed a CART model as a knowledge-discovery tool. We decided to model our CART with just three depth levels in order to produce a straightforward and compact decision tree. Patients selected for CART analysis to be related with RTOG 2 acute toxicity had melanin indices greater than 99. The treatment fractionation, breast cavity volume, and surgical cavity volume all contributed significantly to further increase the classification rate among the patients. The CART model provided an excellent diagnostic performance of AUC=0.959. Because of this high classification accuracy, we think this method could be a valuable adjunct tool for radio-oncologists when prescribing effective treatment options.

Other non-invasive approaches have been employed to study radiation-induced skin toxicity. Laser Doppler flowmetry, for example, has been used to quantify skin toxicity by monitoring microscopic changes in blood flow associated with skin reactions (42). Thermal imaging can detect variations in body surface temperature caused by physiological changes associated to radiation-induced dermatitis. Quantitative thermal imaging markers obtained in the first treatment fractions were used in supervised ML to develop a predictive model for radiation dermatitis (13). Although both Laser Doppler flowmetry and thermal imaging are interesting methodologies, spectrophotometry constitutes a more practical and cost-effective imaging method, because of its practical and intuitive use and the easy explainability of the obtained information’s about the skin changes during the radiation course.

### Strengths and limitations

Deep learning techniques, in particular neural networks, have a relatively high model capacity compared to the machine learning techniques used in this study but clinicians may hesitate to apply prediction models based on black box algorithms difficult to understand. On the contrary, advantages in using machine learning algorithms as LR, SVM or CART are their explainability and interpretability, i.e. their ability to associate a cause to an effect and to justify the results. In particular, we showed how CART analysis was able to identify and construct a binary decision tree for which the correct classification of patient’s toxicity is known. Each path through the tree was defined by a series of dichotomous splits, specifying the values of covariates that lead to a most probable toxicity class. This way, the tree might be viewed as a series of rules that can be used for unknown observations to predict the toxicity class membership. This ability to be easily interpretable makes its implementation into the clinical workflow particularly simple.

A potential limitation of the present study is the number of patients that may affects the power of the prediction model and/or may lead to an over fitted prediction model. To solve this issue, a 5-times repeated holdout technique was performed to provide an internal validation and reduce sample bias. In any case, the created model needs to be externally validated using a distinct dataset in order to assess the prediction model’s consistency and generalizability to new and different patients.

## Conclusions

Spectrophotometry is a simple and accessible technology able to support clinical decisions before a radiotherapy course for breast cancer. Pre-treatment quantitative assessments of skin melanin and erythema could support early clinical management of treatment-related cutaneous adverse effects. CART analysis classified patients with melanin index ≥ 99 and larger breast volume to be associated with RTOG ≥ 2 toxicity with a diagnostic performance of AUC=0.959. On the basis of this study, the radiation oncologist can predict the occurring of acute skin toxicity in selected patients and adjust the treatment pathway by considering alternative treatment options such as a change of fractionation schedule. In the future, spectrophotometric markers may could be beneficial also in other cancer sites, such as head-and-neck where grade 3 toxicity is not uncommon. The knowledge of these potential predictors could improve the management strategies at the beginning of treatment course, thus helping tailoring therapies to reduce toxicities.

## Data availability statement

The raw data supporting the conclusions of this article will be made available by the authors, without undue reservation.

## Ethics statement

Ethical review and approval was not required for the study on human participants in accordance with the local legislation and institutional requirements. The patients/participants provided their written informed consent to participate in this study.

## Author contributions

SC conceived and designed the study, SC, GM and FD draft the manuscript, CR planned the treatments, FB and ADCu collected the data, SC and ADCa performed the statistical analysis, MBo, DP and MBu analyzed the data and wrote the manuscript, LI, CC, MD and AM critically reviewed the manuscript. All authors contributed to the article and approved the submitted version.
